# Studying harms of interventions with an equity lens in randomized trials

**DOI:** 10.1186/s13063-024-08239-x

**Published:** 2024-06-21

**Authors:** Tianjing Li, Evan Mayo-Wilson, Daniel Shaughnessy, Riaz Qureshi

**Affiliations:** 1 Department of Ophthalmology, University of Colorado Anschutz Medical Campus, Aurora, CO USA; 2 Department of Epidemiology, Colorado School of Public Health, Aurora, CO USA; 3 Department of Epidemiology, University of North Carolina, Gillings School of Global Public Health, Chapel Hill, NC USA

**Keywords:** Equity, Health equity, Disparity, Harms, Randomized trials

## Abstract

Equity and health equity are fundamental pillars in fostering a just and inclusive society. While equity underscores fairness in resource allocation and opportunity, health equity aims to eradicate avoidable health disparities among social groups. The concept of harms in interventions—undesirable consequences associated with the use of interventions—often varies across populations due to biological and social factors, necessitating a nuanced understanding. An equity lens reveals disparities in harm distribution, urging researchers and policymakers to address these differences in their decision-making processes. Furthermore, interventions, even well-intentioned ones, can inadvertently exacerbate disparities, emphasizing the need for comprehensive harm assessment. Integrating equity considerations in research practices and trial methodologies, through study design or through practices such as inclusive participant recruitment, is pivotal in advancing health equity. By prioritizing interventions that address disparities and ensuring inclusivity in research, we can foster a more equitable healthcare system.

## Understanding equity, health equity, and harms of interventions

Equity and health equity are intricately connected concepts that are vital in creating a just and inclusive society [1]. At its essence, equity embodies the fundamental principle of fairness, emphasizing the fair and just distribution of resources, opportunities, and outcomes [2]. Equity means that everyone should have an equal opportunity to thrive and succeed irrespective of their background [1, 3]. Health equity extends beyond mere access to healthcare: it focuses on eradicating unfair and avoidable differences in health between social groups [4]. The COVID-19 pandemic starkly revealed health inequities, with poorer people, low-wage essential workers, racialized minorities, migrants, and people experiencing homelessness facing increased morbidity and mortality from the virus [5, 6].

Harms of interventions, the opposite of benefits, refer to undesirable consequences associated with the use of interventions [7]. Across different types of interventions and settings, harms might be described using diverse terms such as “adverse events,” “adverse effects,” “adverse drug events,” “adverse reactions,” “adverse drug reactions,” “risks,” “safety,” “toxicity,” “complications,” or “side effects” [8]. Although these terms are often used interchangeably, they sometimes carry distinct meanings, especially in regulatory research. To illustrate, “adverse events” need not be causally related to an intervention and are always negative, whereas “adverse effects” are also negative but are causally related to an intervention [9]. Meanwhile, “side effects” are related to the intervention and can refer to either negative or positive outcomes [10].

The effects of interventions, including both benefits and harms, can often be distributed unequally across populations. Whether stemming from biological or social factors, differential effects can exacerbate existing inequities or widen disparities across various socio-economic or health dimensions. If one group experiences more benefits or harms than other groups, or if one group bears a disproportionate burden in accessing an intervention, resulting disparities can lead to personal- and societal-level inequities. We believe that such inequities and unintended consequences should be recognized as potential harms of interventions.

## Applying an equity lens to studying intervention harms

An “equity” lens centers around fairness rather than mere distribution. Harms are not distributed equally across diverse populations for both biological and social reasons [11–13]. For example, women, particularly those of postmenopausal age, are more susceptible than men to myopathy (muscle pain or weakness) associated with statin therapy, a commonly prescribed medication for lowering cholesterol [14, 15]. As another example, regulators warn that mRNA platform COVID-19 vaccines increase risk of myocarditis, especially in male adolescents [16, 17]. Biologically driven differences in risks are not inherently equity issues and might not be rectified by addressing social inequities. Nevertheless, viewing harms through an equity lens reflects an ethical imperative to treat all individuals with fairness and respect, emphasizing the obligation to thoroughly study and effectively communicate the benefits and harms of interventions across the various groups that make up the overall population.

Equity problems can arise when researchers neglect to measure and to communicate these differences in harms, or when decision-makers fail to consider these differences when formulating prescribing decisions, clinical guidelines, and reimbursement policies. Notably, researchers and decision-makers often focus on population average effects and overlook the reality that some individuals experience disproportionate benefits while others bear disproportionate harms. Furthermore, achieving health equity necessitates that individuals can make informed decisions by considering the potential benefits and harms relevant to their circumstances. When information about the distribution of benefits and harms across populations is unavailable, such as during earlier stages of intervention development, acknowledging the evidence gaps and outlining future research needs becomes invaluable. Transparency in what we know and do not know should be seen as a strength and can help bolster public confidence in medical and health research.

Furthermore, as we strive to address equity issues, it is crucial to consider the potential unintended consequences and harms arising from implementing well-intentioned interventions, including public health interventions [18–22]. Bonell et al. discuss how public health interventions, even those with positive intentions, can lead to harmful outcomes if not properly evaluated for their ‘dark logic’ or the often negative consequences that arise from the complex interplay of society and human behavior. [23] This concept underscores the importance of anticipating and evaluating the potential harms of interventions, particularly those that might disproportionately affect vulnerable populations [23]. For instance, public health measures aimed at mitigating COVID-19 exposure, transmission, and mortality—such as physical distancing, targeted closures, stay-at-home orders, avoidance of gatherings, and reduced mobility—resulted in unequal health, social, and economic damages [5, 6]. The impacts of these mitigation strategies were particularly pronounced among already disadvantaged and marginalized populations [6]. Women and individuals with lower educational attainment and socio-economic status experienced disproportionate job losses [6]. Social protection systems have proven inadequate, especially for those already in precarious situations [6]. The pandemic disrupted education, yielding broad social consequences for young people, with the adverse impact being greater among economically disadvantaged children [6]. The pandemic also has amplified gender inequality across various facets of society [6].

In recognizing these disparities, it becomes imperative to remain mindful and vigilant of the potential harms that might arise even from implementing well-intended interventions. Interventions can be evaluated not only for their overall effectiveness but also for their potential to either exacerbate or alleviate existing health inequities. Korenstein et al. advocate for a multidimensional framework to evaluate potential harms of health interventions, emphasizing the need to consider a broad spectrum of harms beyond physical effects, such as psychological distress, social disruption, financial impact, and treatment burden. [24] They encourage consideration of all potential harms, particularly those disproportionately affecting vulnerable populations, aligning with the equity lens principle [24]. Finally, by applying an equity lens, researchers and practitioners can better design and implement interventions that both avoid harm and also actively promote fairness and justice across diverse communities.

Incorporating equity considerations in research can help policymakers promote fairness and address the population’s diverse needs. Using vaccine uptake as an example, numerous studies have shown that elderly individuals face barriers in accessing vaccination centers despite being a high-risk group [25]. Additionally, socio-economic factors such as education level and health literacy influence vaccination coverage [26, 27]. Certain racial or ethnic groups may also experience disparities in vaccination rates due to mistrust [28–30]. By acknowledging and addressing these disparities, policymakers can implement targeted outreach programs and allocate resources effectively for the high-risk groups and specific communities.

## Integrating an equity perspective in studying harms of interventions in randomized trials

Equity in randomized trials requires that researchers recognize and proactively address vulnerabilities within specific populations. For example, trials focusing on tele-screening for diabetic eye diseases regularly target underserved or remote communities, recognizing the access challenges faced by these populations [31–34]. These trials aim to mitigate health disparities associated with delayed detection of diabetic retinopathy by using remote technologies, thus allowing individuals to undergo assessments without the need for physical visits to eye clinics. The ACCESS randomized trial demonstrated that the use of autonomous artificial intelligence for diabetic eye exams significantly increased screening rates in a racially and ethnically diverse youth population, effectively closing care gaps and potentially reducing health disparities [35]. This highlights the significance of incorporating equity considerations into the selection of interventions and research questions that cater to the unique needs of all segments of the population.

Furthermore, trialists should adopt methodologies that possess the potential to account for diverse populations and address their unique susceptibilities in the design and conduct of trials. Standardized approaches have been suggested in collecting and reporting participants’ race, ethnicity, and sex in the USA [36, 37]. Toolkit and extensions of CONSORT and SPIRIT are being developed to enhance inclusion and diversity regarding ethnicity, race, and language [38]. The PROGRESS-Plus framework (place, race/ethnicity/culture/language, occupation, out of work, gender and sex, religion, education, socioeconomic status, social capital, age, disability), initially proposed in 2003 and subsequently expanded in 2014, can be useful to assist with the identification and classification of equity-relevant data [39, 40]. However, these guidelines do not guarantee the collection, analysis, and reporting of data from diverse participants, nor do they cover all sources of disparities. For example, an analysis of 200 equity-relevant studies found that a median of 4 (interquartile range = 2) PROGRESS-Plus items were reported in the included studies [41]. The situation is expected to be even worse in non-equity-related studies.

There are sometimes valid scientific reasons to restrict study populations to homogeneous groups. Nonetheless, trialists should be careful to avoid overly restrictive eligibility particularly in later phases of intervention development (e.g., Phase III and Phase IV drug trials, and trials of implementation). Trials that prioritize pragmatic aims over explanatory ones will offer broader generalizability to real-world practice once an intervention’s initial efficacy and harms have been established and it undergoes widespread testing and distribution. In the evaluation of public health interventions, Bonell et al. emphasize the importance of incorporating “realist” principles, which focus on the “how” and “why” by delving into the interplay between intervention mechanisms and their implementation context. [42] Such an approach enables the exploration of interventions’ differential effects across demographic and socio-economic groups, offering nuanced insights for equity-focused policies and practices [42].

To achieve these objectives, trialists can foster inclusivity by engaging directly with communities through strategic partnerships with local organizations and leaders, thereby building credibility and trust [43]. At the outset of designing an intervention and developing a trial, the application of systematic and scientific methods to elicit stakeholder preferences about benefits and harms is critical. By actively incorporating representative voices of the intended users of interventions, researchers can mitigate the risk of unforeseen factors leading to inequities among the subgroups. Trialists also could consider using study designs that address or measure equity issues (e.g., pragmatic trial designs, embedded trials in health systems), harnessing cultural competence among research staff, tailoring recruitment messages to resonate with cultural values, flexible scheduling, offering options for remote participation, use of patient navigators, simplified consent forms, and providing equitable compensation for participants' time and expenses, all of which can serve to diminish barriers to entry [44–46]. Trial process evaluations can also provide fresh insights into intervention implementation, context, and mechanisms of action. For example, process evaluations can identify barriers and facilitators to participation and adherence among different subgroups, and whether interventions are being delivered as intended across various demographic groups. Finally, even trials involving relatively homogeneous populations can collect and report key equity-relevant data to help users understand the applicability of their findings. 

It is important to recognize that the above recommendations pertain not only to harmful outcomes but also to beneficial ones. Additionally, they extend beyond randomized trials to encompass other types of study designs (e.g., observational studies) that are essential for understanding rare or delayed harms.

In conclusion, while not all research is explicitly focused on equity, most research can be designed to promote equity more effectively. Integrating an equity perspective into the study of harms is essential for advancing health equity and promoting fairness in healthcare outcomes. By prioritizing interventions that address health disparities and ensuring inclusivity in research practices, we can work towards creating a more equitable healthcare system for all.

## Data Availability

Not applicable.
